# Supplementary material for: End Sequence Analysis Toolkit (ESAT) expands the extractable information from single-cell RNA-seq data
Source: Genome Res. 2016 Oct;26(10):1397–410. doi: 10.1101/gr.207902.116 (PMC5052061; doi:10.1101/gr.207902.116)
Supplement: Supplemental Material [file supp_gr.207902.116_Supp_Materials.tar.gz › ESAT-esat_v0.1/doc/html/classumms_1_1core_1_1annotation_1_1_compound_interval-members.html]

ESAT: Member List


ESAT


- Main Page
- Related Pages
- Packages
- Classes
- Files

- Class List
- Class Index
- Class Hierarchy
- Class Members

All Classes Namespaces Files Functions Variables Enumerator Pages

- umms
- core
- annotation
- CompoundInterval

umms.core.annotation.CompoundInterval Member List

This is the complete list of members for umms.core.annotation.CompoundInterval, including all inherited members.

|  |  |  |
| --- | --- | --- |
| addInterval(SingleInterval newInterval) | umms.core.annotation.CompoundInterval |  |
| addInterval(int start, int end) | umms.core.annotation.CompoundInterval |  |
| compareTo(CompoundInterval other) | umms.core.annotation.CompoundInterval |  |
| complement() | umms.core.annotation.CompoundInterval |  |
| complement(int start, int end) | umms.core.annotation.CompoundInterval |  |
| CompoundInterval() | umms.core.annotation.CompoundInterval |  |
| CompoundInterval(int start, int end) | umms.core.annotation.CompoundInterval |  |
| CompoundInterval(CompoundInterval other) | umms.core.annotation.CompoundInterval |  |
| contains(CompoundInterval other) | umms.core.annotation.CompoundInterval |  |
| containsExactInterval(SingleInterval i) | umms.core.annotation.CompoundInterval |  |
| containsInterval(SingleInterval i) | umms.core.annotation.CompoundInterval |  |
| equals(CompoundInterval other) | umms.core.annotation.CompoundInterval |  |
| getBlocks() | umms.core.annotation.CompoundInterval |  |
| getCoordinateAtPosition(int positionInInterval) | umms.core.annotation.CompoundInterval |  |
| getEnd() | umms.core.annotation.CompoundInterval |  |
| getPositionAtCoordinate(int coordinateInReference) | umms.core.annotation.CompoundInterval |  |
| getSpan() | umms.core.annotation.CompoundInterval |  |
| getStart() | umms.core.annotation.CompoundInterval |  |
| intersect(CompoundInterval other) | umms.core.annotation.CompoundInterval |  |
| length() | umms.core.annotation.CompoundInterval |  |
| minus(CompoundInterval other) | umms.core.annotation.CompoundInterval |  |
| moveToCoordinate(int coordinateInReference) | umms.core.annotation.CompoundInterval |  |
| numBlocks() | umms.core.annotation.CompoundInterval |  |
| overlaps(CompoundInterval other) | umms.core.annotation.CompoundInterval |  |
| overlaps(CompoundInterval other, int buffer, boolean ignoreBlocks) | umms.core.annotation.CompoundInterval |  |
| overlaps(CompoundInterval other, boolean ignoreBlocks) | umms.core.annotation.CompoundInterval |  |
| overlaps(CompoundInterval other, int buffer) | umms.core.annotation.CompoundInterval |  |
| overlaps(SingleInterval interval) | umms.core.annotation.CompoundInterval |  |
| overlaps(SingleInterval interval, int buffer) | umms.core.annotation.CompoundInterval |  |
| removeInterval(SingleInterval i) | umms.core.annotation.CompoundInterval |  |
| setEnd(int end) | umms.core.annotation.CompoundInterval |  |
| setStart(int start) | umms.core.annotation.CompoundInterval |  |
| shift(int delta) | umms.core.annotation.CompoundInterval |  |
| toString() | umms.core.annotation.CompoundInterval |  |
| union(CompoundInterval other) | umms.core.annotation.CompoundInterval |  |


---

Generated on Fri Aug 15 2014 15:11:08 for ESAT by  

 1.8.7
